# Bibliographical Notices

**Published:** 1855-12

**Authors:** 


### BIBLIOGRAPHICAL NOTICES.

*A Treatise on Medical Jurisprudence.* By FRANCIS WHARTON, Author of "A Treatise on American Criminal Law," etc., etc., and MORETON STILLÉ, M. D., Lecturer on the Principles and Practice of Medicine in the Philadelphia Association for Medical Instruction. Philadelphia: Kay & Brother. 1855. 8vo. pp. 815.

Medical Jurisprudence, "the science which teaches the application of every branch of medical knowledge to the purposes of the law," would seem to be a subject of trivial importance were we to estimate its value by the little time and attention generally bestowed upon its study. Its claims upon the profession are nevertheless among the very strongest. We grant that its skilful or inefficient exercise may not affect the community so directly for good or evil as may that of some of the other accomplishments of a medical education. Still, it can not be denied that the practitioner of medicine is at all times liable to be called upon for his special evidence in the elucidation of questions which come home to the business and bosoms of individuals and communities as nearly as life and health, and in which every thing may depend upon his acquirements in the kind of training which medical jurisprudence alone can adequately give.

The presumed and actual ignorance of juries of all professional knowledge, as a matter of course, inclines them to confide in the testimony of an expert, who is capable of conveying clearly and precisely the information looked for, and who is able to maintain his position, whether as to fact or opinion, in the face of an astute and learned cross-examination. On the other hand, for the same reason, nothing is more lame and hopeless in its practical influence on the witness stand, or destructive to the moral weight of medical authority, than the blundering self-contradictions, hesitation and confusion, not to say positive stupidity as to the point at issue, with which even eminent members of our fraternity too often annoy the court and mistify an already puzzled jury. How often might we escape these humiliating exhibitions, and how easily repel the sneer which is only too well earned by many otherwise deserving members of an unappreciated calling, if such works as the excellent one before us were more frequently consulted! How much better for us all were they made the text books to a bonâ fide course of teaching in our regular curriculum, instead of being left, as they almost universally are, to the caprice of the student, as matters of mere elegant amusement.

Our readers can be at no loss to recall notorious as well as minor instances of the wretched consequences of a want of this kind of medical instruction, or of an amount of self-preparation which the treatise of Wharton and Stillé abundantly provides for and brings within the reach of every intelligent practitioner of law or of medicine.

There are men of high standing in each vocation who are disposed to regard the position here assumed for the study of medical evidence as unduly exalted. They wish to deny to it the name of a distinct department of science, and accuse it of increasing the very difficulty and embarrassment which it was intended to do away. The most conclusive answer to such objections would be rendered, as it has been time and time again, by the argumentum ad hominem in a court of justice. We suspect that few who have tried it in such a spirit will deny the truth of Mr. Taylor's remark, when he says that he never knew such

"indifference manifested by one who had once been summoned as a medical witness to a court of law."

But it is to the doubting members of the two professions that we wish especially to recommend the book before us, as one which is, of all others, the most likely to satisfy their scruples, if not to change their views. It will command their attention as the joint production of able representatives of either body. It meets the most urgent want of sceptics of both sides, inasmuch as each of the two authors was peculiarly well qualified by character of mind and by long previous study, not only to discuss lucidly the parts assigned to him, but to assist and guide his fellow in the duty of co-ordinating the different topics—of giving to each point of law or medicine, respectively, the cast required to make it useful and acceptable alike to lawyer and physician.

The best idea, however, of their objects and the general arrangement of their labors, may be derived from the surviving author's preface, two paragraphs of which we here subjoin.

"The two points which were mainly before the authors of the following treatise when they entered upon its preparation, and the hope of reaching which formed their chief inducement in approaching a topic which has already been in other respects so ably and fully discussed elsewhere, were first, the incorporation in its pages of the results of late continental, and particularly French and German research; and secondly, the bringing together stereoscopically—if the metaphor can be permitted—of the Legal and Medical points of vision, so that the information required by each profession might be collected and viewed at the same time and within the same compass. It was felt that in the usual range of medico-legal exposition, there was a great deal that, though interesting to the medical man, is unnecessary to the legal practitioner; and, on the other hand, it is equally clear that there are many points upon which the latter needs information, which the former, either from inadvertence or what would be to him their extreme simplicity, may forbear to touch. The converse also is true, viz., that the legal writer who undertakes such a work, except in subordination to medical advice, may exhibit very satisfactorily the necessities of legal practice, but will fail to supply the information by which these necessities can be met.'

"These two points, viz., the absorption of recent medico-legal research in France and Germany, and the union of the medical and legal stand points, it was here hoped to reach, not so much by a concurrent authorship of each page, as by a general preliminary comparison of views and adjustment of material by the two writers by whom the task was undertaken, followed up by a division of the subject matter between them in subordination to the plan previously agreed upon. In pursuance of this scheme, the second, third, fourth, and fifth books,—

viz., those on the Fœtus and New-born child, on Sexual Relations, on Identity, and on the Causes of Death, were assigned to Dr. Stillé, with the exception of those sections in the two former which concern the legal relations of gestation, abortion and rape; while to the present writer fell the preparation of the first and sixth books, embracing Mental Unsoundness and the Legal Relations of Homicide, together with the general disquisition on Indicatory Evidence with which the latter book concludes."

The first book, containing two hundred and twenty-eight pages, is devoted to the consideration of Mental Unsoundness in its legal relations and interpretation. This portion belongs, as above stated, and as is already well known, entirely to Mr. Wharton. It was published separately some time ago, and has attracted a great deal of well merited attention. We have had the pleasure of noticing it at length in our number for August of this year, and therefore shall not dwell upon it now.

With regard to the technical merits of the other portions of the volume by the same author, we can not pretend to say much in a purely medical notice. We have been greatly interested, however, in the clear and ample expositions, and in the admirably concise and convenient method of arrangement and illustration, which characterize the chapters on the legal relations of rape and of homicide, fœticide and infanticide. The discussion of each of these topics is very materially aided and simplified by a preliminary analysis. These analytical tables are among the most striking and valuable features of the book, and will doubtless prove particularly serviceable to the medical inquirer. Some idea of their scope and tendency may be derived from the following brief extract, which introduces the article on the "Legal Relations of Rape."

§ "457. The points to which medical testimony is most likely to be invited, in prosecutions for rape, are the following:—

1st. Submission of prosecutrix.
- (1) From artificial stupefaction.
- (2) From ignorance of the nature of the act.
- (3) From mistake of person.
- (4) From fear.

2d. Prior want of character of prosecutrix.
3d. Subsequent suppression of the fact by prosecutrix.
4th. Extent to which coition was carried.
5th. Want of age of defendant.
6th. Want of sexual capacity of defendant.

The law on each of these points, will be now briefly considered.

1st. *Submission of prosecutrix.*

This may happen from either of the following causes:—

(1) *From artificial stupefaction.**

"§458. It makes no matter whether the drug was given for the purpose of producing stupefaction, in order that the rape might be effected on the female thus made unconscious, or whether it was administered for the purpose of causing sexual excitement, and thereby leading to a voluntary submission. It is rape in either case; the law being, that the overcoming of chastity, and the destroying of resistance by artificial means, is rape, when the offence is consummated. If the result of the dose be stupefaction, and if on the woman, thus become insensible, carnal intercourse be effected; it is rape, though the intention was merely to excite. Thus, where the prosecutrix was made drunk by the prisoner, who then violated her person, it was held in England, where, from the offence being capital, it is kept within its strict common law limits, that the crime was rape, though the jury expressly found that the liquor was given with the intent of *stupefying* not *exciting.*† And in this case, it was held, that where the insensibility is the defendant's act, and where the defendant knows that 'the act was against the prosecutrix's consent at the last moment she was capable of exercising her will,' it is rape. On this point agreed all the ten judges of England, constituting the final Court of Revision in criminal causes; and it was not required by the exigencies of the case that they should go further. Several, however, thought—and this view is in accordance with the analogous cases to be hereafter noticed—that the crime was consummated by the mere act of knowingly violating an insensible woman, whether the insensibility was produced by the defendant himself or not.

"§459. In the late prosecution of Dr. Beale, in Philadelphia, which has been noticed above,‡ the point was not made, and it was assumed by both sides, that carnal intercourse with a woman who was stupefied by chloroform was rape, though the chloroform was administered, at her request, for the purpose of facilitating the extracting a tooth. And if the law—and the cases to be subsequently noticed unite with the reason of the case in indicating that it is—it goes to establish the broad position, that rape is sexual intercourse with a woman, not *against*—as has been formerly said—her will, but *without* it." Pp. 351, 352.

Under the head of Corpus Delicti a great variety of deeply absorbing cases, new, as well as old, are presented in a most instructive manner. It would be easy to attract the most indifferent reader with passages from these strange annals of crime and its

* See the medical relations of this point, *ante*, § 439.

† R. *v* Camplin, 1 Car & K. 746; Wh. Cr. Law, (3d ed.) 514–15.

‡ See *ante*, § 445.

marvellous exposure. A quotation of a different kind, however, will serve our purpose better. It is in relation to "Marks of Violence," and may be presented as a pretty fair example, in its way, of the manner in which our author indicates the course to be pursued by medical investigators and observers.

"§ 1138. Marks of violence, in connection with the cause of death, have already been considered. At present they are only to be noticed in connection with the question of intent. It cannot be doubted that when a wound is found to have been inflicted in a secret or concealed part, which is inaccessible in sudden and passionate conflict, it bears a violent presumption of having been the result of design. Thus the wounds of which the Scotch historian tells as having been inflicted by forcing a heated iron into the fundament, could have been explained in no other way than on the hypothesis that to death was intended to be added concealment. In the same class may be enumerated the thrusting of a needle in the navel of an infant, running a sharp but slight intrument in the cavity behind the ear, dropping corrosive acids into the ear itself, and forcing molten lead down the throat through a tube; of each of which resorts the books give instances.* The principle on which the presumption of intent can be drawn from such cases is, that a person acting under the impulse of passion is much less likely to inflict a skilful wound, than one whose act is the result of premeditation.†

"§ 1139. Whether the wound was inflicted in self-defence or otherwise; whether it was self-inflicted, or inflicted by a stranger; whether the perpetrator of the crime was an expert or otherwise,—may also be deduced from the wound. And the direction of the wound may often be shown for the purpose of testing the validity of a defence. Thus, where the defence was, that the ground being rough and slippery, the prisoner stumbled, and both barrels of the gun had gone off by accident, the defence was confirmed by tracing the direction of the shot, which was found to be pointed upwards.‡ The difference in appearance between wounds inflicted before and after death, has been already considered.‖

"§ 1140. It is by medical testimony alone that the agency of the alleged violence, as a cause of death, is to be determined; and if the death was not accelerated by such violence, the defendant must be acquitted. Thus, in 1847, on a trial for manslaughter, the surgeon who had attended the deceased, stated that on examining her body he had

* Mittermaier von Beweise, 402; Demme's Annalen des Criminalrechts, vol. iii. p. 215; Bauer, Theorie des Anzeigenbeweises; Henke Darstellung, sec. 99; Blanci de Indiciis, Venet, 1545; Reinhardt de eo quod circa reum ex Præsumpt. Convinc. et Cond. Just. &c. Erford, 1732; Heinroth in Hitzig's Zeitschrift, n. 42, p. 257.

† Presumption from gun-shot wound, see *ante*, § 811.

‡ Watson on Homicide, § 246.

‖ *Ante*, § 810--816.

found the mark of an old wound on her head, and a slight bruise on one of her thighs; but he further stated that he made a *post mortem* examination of the body, and that his opinion was, that the cause of the deceased's death was confirmed consumption, her lungs being tuberculous, and that it had not been accelerated by violence, but was wholly attributable to natural causes. The defendant, under the direction of MURPHY, Sergeant, who consulted with Lord Chief Baron POLLOCK, was acquitted.* But it is no defence that the deceased was laboring under a mortal disease, if death was accelerated by the defendant's violence;† and this, no matter how remote the cause, if the intention was to commit an assault, and death resulted."‡ Pp. 748, 749.

Book second, which is the first of those assigned to Dr. Stillé, is divided into six chapters. These are occupied respectively and successively with the "Signs of Pregnancy," "Delivery," "Duration of Pregnancy," "Superfœtation," "Abortion or Fœticide," and "Infanticide." The third book is divided into three chapters, which treat of "Doubtful Sex," "Sexual Disability" and "Rape." Book fourth contains but one chapter, which is devoted to the "Identification of the Living or Dead."

The fifth and last book of the medical series, discusses the "Questions Relative to the Causes of Death." It is divided into two parts, the first of which includes "Poisoning" and the various poisons, in numerous chapters under the usual heads. The second of these parts continues the course with the consideration of the "Other Forms of Violent Death," such as Wounds, Burns, Spontaneous Combustion, Heat, Sun Stroke, Lightning, Cold, Starvation, Suffocation, Strangulation, Hanging and Drowning, and completes it with a chapter on "Medico-Legal Examinations."

Even a slight examination of these pages will satisfy those who are familiar with the subject, that the promise of the preface is abundantly sustained in them, no less than in the essays of his accomplished colleague. Indeed the greater scope and variety of available material in the French and German publications required by Dr. Stillé, has enabled him to render the peculiarity aimed at in regard to the results of European continental research, especially remarkable in his share of the labor. Nearly every page gives proof of the industry and discrimination with

* R. *v.* Conner, 2 C. and K. 518. See also R. *v.* Crompton, C. and Mars. 597
† State *v.* Morea, 2 Ala. 275. ‡ Wh. C. L. (3d edit.) 363.

which he has explored and gleaned from those invaluable and ever-growing fields of scientific cultivation, which are beyond the reach of ordinary students. But in thus gathering and presenting to his American readers a digest of experience, which, although hitherto comparatively little known, is unsurpassed in authority and varied interest, he has by no means lost sight of British and American cases and results. The cream of the whole subject is laid before us in a manner at once simple and unpretending, yet with a brevity and logical precision, an unusual cautiousness, and a judicious and manly independence of opinion, which decidedly increase the authority of the book, while they add to its freshness and interest as a truly original production. As far as we are able to determine, it is perfectly reliable in its doctrine and citations, and may be said to have been brought, in some of its cases and recently established practice, in advance of any other of the standard manuals yet published in our language. We shall have an opportunity to recur again to the history as well as the peculiar features of the essay of our departed friend, which render it a sadly cherished memento of a career so full of promise and so painfully cut short in the act of its fulfilment. Meanwhile we take pleasure in submitting a few chance extracts which may serve as examples of his ordinary style and mode of handling his topics. In relation to quickening in pregnancy, he dwells upon an important but still unappreciated truth in the following language:

"§ 283. The undue importance attached to quickening, from the earliest times, arose from an error which modern science would long since have consigned to oblivion, had it not been fatally incorporated into the laws of various countries. It was supposed that the fœtus became endowed with vitality at a variable epoch after conception, and that quickening was an indication of the moment at which it became thus animated. Such an error, explicable in the infancy of physiological science, by an inadequate knowledge of the development of the embryo, confirmed by absurd ecclesiastical canons, and handed down from one criminal code to another, should now, when ignorance is no longer excusable, disappear from our penal system. To whatever cause the act of quickening may be attributed, its explanation is not dependent upon a solution of the question relative to the precise moment at which the child becomes endowed with life. If it be due to the first motions of the child, perceptible to the mother, it is merely an indication of its *first muscular movements;* and if it is caused by the sudden rising of the uterus from the pelvis, it evidently has a still more distant connection with the phenomena of life. No serious argument is required to prove that the fœtus, in its embryonic condition, is a new being, living by its connection with its mother, dying when this is destroyed. However rudimentary its form, it is not an inorganic body, constituted by the casual aggregation of atoms, but a living creature, from whose undeveloped lineaments a perfect human shape is to be evolved. A pulsating heart, and a nervous tract, are among its earliest recognizable elements. Reason and observation equally declare its essential vitality." P. 232.

The following curious history of a case of feigned delivery, reminds us of the somewhat similar, but more honest, management of the barren wife of a patriarch of old.

"§ 301. *Feigned delivery.* Delivery may be feigned from a variety of motives, into which it is not necessary for us to enter. A medical inspection can hardly fail to expose the deceit, and usually the collateral proof is sufficient. We have abridged the following case of feigned delivery, on account of the wonderful ingenuity with which the imposture was conducted. Dr. Albert relates that he was called upon to see a poor girl of twenty-one years of age in her last illness. In the presence of the physician and clergyman of the district, she gave the following narrative and confession. Some eighteen months previously she had entered the service of a married couple as housemaid. Her master, who was young and handsome, and assumed the title of Baron, had no children. He succeeded, by tempting presents, in overcoming her virtue. He then represented to her, that an important inheritance depended upon his having an heir, but having been married five years, and his wife still proving unfruitful, he had no longer any hope of having children by her. He then proposed to the girl, that in case she should prove with child, and would allow him to cause it to appear as his own legitimate offspring, he would not only give her a considerable sum of money, but would also let her remain in the house with her mistress, in order that she might be always near her child. She accepted the proposal, and as soon as she found herself to be pregnant, the preparations were made to carry out the projected imposture. The girl remained in the house, living in the most retired manner, while her mistress played the part of a lady in an interesting condition. She introduced wool and folded napkins under her dress, and thus gradually let her rotundity become apparent, rubbed her breasts frequently, in order to develop them, fainted in church, was often ailing and sent for midwives and consulted them concerning her symptoms; physicians were also called upon, and every means taken to make public her happy expectations, so that no one had any suspicion that she was not pregnant. The traces of her monthly sickness were carefully concealed.

"At last, in due time, the young girl fell in labor, which was allowed to advance considerably before the midwife was sent for. In the mean time the bed was arranged in the following manner. A board was taken out of the bottom of the bedstead, and immediately above this opening a hole was made through the mattress and paillasse large enough to allow the legs of a person to pass through and rest upon the floor. The bed was made in such a manner as to sink down towards the headboard, while it was elevated below the opening in the mattress. The mistress now leaned in a sitting position, with her legs through the opening in the bed, and supported against the headboard, while the servant lay across her lap on a feather bed, in the attitude of labor. Her body was entirely concealed by the bed coverings, which also concealed her mistress up to the neck. The midwife, upon her arrival, found the Baroness, as she supposed, in the throes of labor; she made the necessary examination, promised a speedy deliverance, and gave the usual words of comfort. The lady, however, screamed lustily at every pain, the approach of which she became conscious of by the involuntary contraction of the poor girl's body; while the latter suppressed her cries as much as possible, except when she could mingle them unperceived with those of her mistress. A living male child was soon born, and the after-birth followed it immediately. While the nurse was busy in washing and dressing the child in another room, the girl escaped from the bed into an adjoining chamber. The Baroness, before the return of the midwife, drew her feet up from the opening, covered it over with the bed, and stretching herself out upon it, forbad the midwife, (who was desirous of ascertaining her condition) to touch her, except to wash off the blood with which she had previously soiled her thighs, declaring that she was in so much pain, that she could not endure the slightest touch. The child was baptized, and on the second day put to the breast of the lady. As, however, very naturally, it found nothing there, the midwife was discharged on the pretext that the Baroness' own attendant could now take care of the child, which immediately upon her departure was confided to its own mother. The remainder of the girl's history not being essential here, is omitted. Unexplained circumstances prevented the fraud from succeeding. The authors of the conspiracy fled, leaving the servant-girl sick and in a state of destitution. She died, from the effects of privation and exposure, shortly after having made this confession."* Pp. 242, 243.

Under the head of "General Considerations" on wounds, his view of the proper use and meaning of the term "wound" is thus expressed.

"§ 792. The meaning of the term 'wound,' in popular language, can hardly be misunderstood. Its meaning is, indeed, not precise, nor is the term susceptible of an exact definition. It is applied to most forms of bodily injury caused by external violence, apart from any consideration of the means by which this was inflicted, but, in general, involving the idea of a breach of continuity in the skin. This latter element, however, of the definition, is not included in the surgical meaning of the word, since one class of wounds, called 'contused,' and

* Henke's Zeitschrift, Vol. xliv. ·d 172.

recognized by all surgical writers, is not attended necessarily with any division of the skin. Indeed, the popular understanding of the expression appears to be more rational than is the professional one, for if a contusion or bruise can properly be called a wound, it is difficult to perceive why a sprain, fracture, or dislocation, is not equally entitled to the name. Yet these latter injuries are never so called by surgical writers. It is difficult, also, in consequence of the apparent vagueness of this term, to know whether burns and scalds can properly be ranked as 'wounds.' The immediate effect of the application of a burning or heated body to the skin, may not be such as to cause more than a redness of the surface, or an elevation of the cuticle into a blister, but the surface of the skin may afterward, by the giving way of the cuticle, be exposed, and it is then termed 'wounded.' Hence, the reader will perceive, that any legal limitations of the meaning of the words, whether based upon popular or professional definitions, are liable to be erroneous, if the intention be really to designate the results of external violence by a name which shall comprise them all. In treating of this subject in its medical aspect alone, we shall make use of the word *wound* as expressive of any form of bodily injury caused by external violence, since it is only by such course that the medico-legal bearings of the subject can be properly considered. Hence we have used the term WOUNDS as a convenient designation for this chapter, entirely irrespective of the possible surgical or legal limitations of the word."

A very wholesome caution is administered in the paragraphs which follow.

"Whatever parts of this examination call for the application of knowledge of which he may not be possessed, as the use of the microscope, or chemical analysis, should be committed to one who is really an 'expert' in these branches. The idea is much too prevalent, and should be corrected, that the practitioners of medicine must necessarily be acquainted with all the appliances and new modes of investigation which modern science has produced; in other words, that every physician is equally competent to undertake the examination of a case involving the question of homicide. It is to this cause chiefly, viz., the disparity in the attainments of one physician as compared with another, and also to the natural division of medical science and practice into numerous departments, some one of which may be cultivated to the exclusion of others—that the 'disagreement of doctors' is really due. Men of equal medical attainments will rarely differ upon an essential point of pathology or practice, but ignorance, or a defective knowledge in medicine, does not differ from that in any other branch of science in being usually associated with presumption and obstinacy. Still there are few practitioners of medicine who are thoroughly prepared to enter upon an examination of all the medical aspects of a case of violent death; familiarity with the means required to carry through such an investigation, can be gained only by special study, for which, to the majority, time is wanting.

Circumstances may, however, impose upon the physician the duty of making an examination for which he does not feel himself fully competent. In remote or interior parts of the country the means for the successful prosecution of a medico-legal inquiry are usually not at hand; who ever may be obliged to undertake an examination under such circumstances, should endeavor to obtain the assistance of a colleague, and should candidly represent to the authorities the necessary imperfection of the examination, and what influence this may have upon the objects of the inquiry." Pp. 540, 541.

Again in regard to suicidal wounds.

"§ 818. That part of the *circumstantial evidence* which requires medical knowledge for its elucidation, is often most curious and important, and as it has to deal with conditions incessantly varying, and is founded upon no familiar principles, nor positive scientific basis, but rather upon loose and badly observed facts, must partake of the same nature, and often appear discordant and improbable. Each medical witness may put together in a different manner, the materials with which he is required to reconstruct the scene immediately preceding death; and a successful result will most certainly reward him, who with the most acute perception unites the largest and most familiar acquaintance with similar facts. In estimating the probabilities in reference to the manner of death, the physician has need of all the aid which a general observation of the workings of the human mind can afford him; his psychological knowledge and his medical experience must here go hand in hand, for it is his task and duty to offer an explanation of the mutual dependence of motives and results, and that, in the same disinterested and merely scientific manner, that would be required by the demonstration of any curious fact in physics." P. 555.

The question of responsibility in the surgical treatment of wounds is well discussed.

"§ 844. Death, indeed, sometimes takes place during or immediately after surgical operations undertaken for the relief of the wounded person. The question of responsibility in this case, belongs to the legal portion of the subject. It may not, however, be out of place to remark that the surgeon can seldom foresee, with confidence, the issue of capital operations, for there are many individual peculiarities and causes beyond his control, which may make it unfavorable. The same may be said of any plan of treatment, whether it involves a serious operation or not. The question may arise, whether the surgical treatment employed was the best that could be devised, and whether, had some other course been pursued, a favorable result might not have been obtained. Or, it may be alleged that the treatment was so unskilful, or the patient so much neglected, as to be the occasion of the fatal termination of the injury. That these facts should be established beyond dispute, it ought to be shown that the treatment was marked by the omission of something universally recognized as of primary importance. But as every surgeon has some peculiarities in his practice, and as the mode of treatment of bodily injuries, from the progressive nature of the medical art, is various, this omission should be looked for only in those points which betray an ignorance of the fundamental principles of surgery. However much the opinions of competent persons may differ respecting the choice of remedial means, they will generally, we think, be found united upon the principles which should govern their application. Still, occasionally, the plan of treatment may be so singular, although apparently founded upon correct notions of the curative process, as to call for reprobation. Thus, in a case which occurred in Saxony, a surgeon was deprived of the liberty of practising his profession in that country for having attempted to promote bony union between the fragments of a fractured patella, by the novel expedient of firing a pistol between them. Although no permanent injury was done to the patient, who, indeed, a few months after the operation, declared that his leg was nearly as good as the other one, and that he was even able to dance and to walk long distances, yet the medical commission charged with the case very properly considered the operation as likely to prove a dangerous precedent if it were not condemned.*

"§ 845. The difficulty is not so great where the original wound has been trifling, chiefly because its comparatively innocuous character can be clearly shown. Thus, for instance, if the hand have been wounded and one of the arteries divided, compression may be necessary to arrest the hemorrhage. But if a surgeon, with this view, should apply a bandage so firmly, or on the other hand, leave it on so long as to cause mortification of the part, and death ensue in consequence, it is evident that the treatment has not only been unskilful, but that it has really been the cause of death, since the wound of the hand was neither, in itself, mortal, nor would it have produced death in the manner described. But, in severe injuries, in which various complications arise and require the exercise of the greatest skill that learning and experience can give, it cannot be expected that some will not terminate fatally, which, perhaps, under more favorable circumstances, or a better plan of treatment, might have had a fortunate issue. The most humble surgeon may chance to receive the charge of an injury which calls for the enlightened tact and experience of a highly educated man; if his treatment should prove unsuccessful, he should be prepared to show, if required, that his patient had the best care which he was able to afford him, and, if possible, that he consulted with one or more colleagues respecting the treatment. In the language of Judge Woodward, 'The implied contract of a physician or surgeon is not to cure—to restore (e. g.) a fractured limb to its natural perfectness, but to treat the case with diligence and skill. . . . He deals not with insensate matter like the stonemason or bricklayer, who can choose their materials and adjust them them according to mathematical lines, but he has a suffering

* Casper's Vierteljahrschrift, 1852, Bd. 1, H. 1.

human being to treat, a nervous system to tranquillize, and a will to regulate and control."* Pp. 569, 570.

The chapter on "Spontaneous Combustion," although brief, is particularly deserving of attention. His belief in the reality of these disputed phenomena is, to some extent, affirmative, as may be seen by the annexed quotation from his opening paragraph.

"Not having adopted a theory with a desire to find those facts only which might be adjusted to it, but desirous of discovering the real extent of our knowledge relative to the phenomenon of what is called spontaneous combustion, we have examined the subject not without some care and earnestness. The result of this investigation has shown us that if there is not such a phenomenon as the actual spontaneous combustion of the human body, there is sufficient evidence to prove, that in some cases it may acquire a præternatural inflammability and that this peculiarity can be recognized by the trifling source of combustion compared with the rapidity and extent of its progress. We do not hesitate also to affirm that a belief in the actual occurrence of the phenomenon referred to may be entertained, without a satisfactory scientific explanation." P. 591.

The subject of medico-legal examinations is introduced with the following admonitions:

"§ 943. The physician who is called upon to make an examination of a person found dead under suspicious circumstances, has devolved upon him a task of no little gravity. He therefore should endeavor to come to it prepared to acquit himself of his duty in such a manner, that he will afterwards not have to regret having imperfectly discharged it. Not only is familiarity with anatomical dissection required, but a far greater carefulness, and a more searching examination than in cases of death from disease, since in these latter, the object of the investigation is into the nature of the morbid cause of death, and the acquisition of greater familiarity with pathological facts. Moreover his attention must be given to many circumstances which, in these, it is not necessary to observe, viz: all those matters which may throw light upon the mode of death, such, for instance as the position of the body in relation to surrounding objects, and the locality in which it is found. The duties of the examiner, and the facts necessary to observe, may be arranged under the following heads:

1st. *Locality.*
2d. *Identity.*
3d. *Indications of violence or unnatural death.*
4th. *Manner of conducting the autopsy.*
5th. *Natural aspect of the organs at different ages.*
6th. *Mode of drawing up Reports.*" Pp. 645, 646.

*McCandless *v* McWha, Error to Common Pleas of Beaver County. Am. Jour. Med. Sci. Jan. 1854, p. 273.

These different heads are dwelt upon at length in a very useful series of instructions, of which the summary of the "*Natural aspect of the internal organs*," is new and particularly desirable as a guide to the post-mortem examiner.

With these few passages from this admirable work we are obliged to close our notice of its valuable contents. We congratulate the publishers on the handsome manner in which they have contributed, in the getting up of the volume, towards the popularity which it so fully deserves, and will doubtless rapidly obtain.

It only remains for us, as the Journal collaborators of Moreton Stillé—his fellow-laborers in other similar paths, and as sorrowing associates who had long since learned to prize his worth,—to say amen with all our hearts to the eminently just and feeling tribute of Mr. Wharton, which he offers in the sadly interesting history of his lamented colleague's last year of study and of its unfortunate and, to us, irreparable end.

"Of the manner in which was performed at least a portion of the task whose history is now given, it may not be unsuitable for the writer who now survives to speak. It was to the preparation of this portion that the last year of Dr. Stillé's short but distinguished professional career was given. It was a year of patient and severe research, marked, to an extent of which the annals of science afford few parallels, by the most self-denying industry, as well as by a rigorous and almost fastidious conscientiousness in the pursuit, not only of truth, but of the most appropriate terms by which that truth could be expressed. And the labor was not one which derived any portion of its severity from the want of prior preparation. Dr. Stillé had not only enjoyed great opportunities for literary and professional culture, but what is rarer, these opportunities were faithfully improved. The liberalizing influences of European culture, as well as the simpler discipline of home instruction, passed not over him in vain. The schools of Europe received from him in the prime of his early manhood the same single and conscientious attention as the schools of Philadelphia in his youth. He found the pleasures of travel and the desultory influences of foreign habits of no more avail in drawing him from a laborious personal attendance on the hospitals—those great repositories of disease which, in the developement of God's wonderful providence, are made the arsenals which supply the weapons by which the maladies that necessitate them are to be combated—than he found the more primitive habits and more limited associations of his native city. The great continental tongues, in their scientific as well as their popular relations, were mastered by him to a completeness of which, among persons of his age, we have rare examples. Few men, even among the most mature, have gone to the grave so richly fraught with the literature of a profession, which to him was a philosophy as well as an art. And few, at so early age, have gone to the grave with faculties under more complete moral discipline. To one of his most remarkable qualities,—that delicate and yet modest firmness of perception which, unwarped on the one side by pride in his own opinion, or on the other by undue deference to that of others, enabled him, after the most difficult and subtile research, not only to reach but to express the truth—no one has had better cause to testify than the present writer. The work which these lines now close was one which brought both of those who engaged in it into the most intimate and affectionate personal intercourse for many months; and the one who survives can now scarcely look back upon the preparation of a single page without having additional cause to remember and record those high mental qualities and culture, whose value in the present case was only increased by the gentleness, the refinement and the fine sense of personal honor with which they were associated.

. . . . . . . .

"Perhaps, indeed, even in a profession whose history has been so marked by acts of zeal and disinterestedness, when we take into consideration the fact that Dr. Stillé was impelled by no other motive than that of professional love and enterprise in the severe course of study and self-sacrifice in which he was engaged, there will be found few cases where these qualities have been so eminently exhibited as the present. Possessed of an ample fortune, he was one of those uncommon instances in which the most arduous and protracted courses of preliminary trial are gone through with under the calm and equal effect of a will which is impelled neither by necessity nor the desire of present applause, but by the faith in a distant future, in which the result will be none the less precious because it is the longer delayed.

"But this future was one to which Dr. Stillé—and to the great loss of popular as well as of medical science—was only in part to arrive. Early in 1855, he received the appointment of Lecturer on the Practice of Medicine in the Philadelphia Association for Medical Instruction, and at the end of June closed the first portion of a course of lectures of which it is not too much to say that they were received with unmixed satisfaction by the class to whom they were addressed, and the colleagues with whom he was associated. In the first week of July he sent from his office the last of the manuscript of that portion of the following pages which fell under his charge, and almost immediately afterwards was stricken down by a disease which found him with strength impaired by the exhausting studies of the preceding winter. On August 20, 1855, he died at Saratoga, almost at the moment when the press was issuing the last sheets of a work which contains so much worthy of being erected as a monument in which his professional brethren will recognize the impress of his high intellectual gifts and culture." Pp. iv. v. vi. of Preface.

*Principles of Human Physiology, with their chief applications to Psychology, Pathology, Therapeutics, Hygiene and Forensic Medicine.* By WILLIAM B. CARPENTER, M. D., F. R. S., F. G. S., Examiner in Physiology and Comparative Anatomy in the University of London, &c., &c. *A new American, from the last London Edition, with two hundred and sixty-one illustrations. Edited, with additions,* by FRANCIS GURNEY SMITH, M. D., Professor of the Institutes of Medicine in the Medical Department of Pennsylvania College, &c. Philadelphia: Blanchard & Lea. 1855.

This is the sixth large edition (as we are informed) of Dr. Carpenter's Principles of Human Physiology, a sufficient proof, were one wanting, of its acceptableness to the student and practitioner. In many of our schools it has become the text book on the "Institutes," taking the place of others that for a long time held undisputed sway. A modification has been introduced into the present volume, however, which we fear may, to a certain extent, mar its usefulness as a companion to the student, though it will probably enhance its value in the eyes of the practitioner. This consists in the carrying out of a plan long entertained by the author, of making three distinct treatises of the Human, Comparative and General Physiologies, transferring to each of these volumes whatever was found in the other relative to the special departments, thus making three "independent but mutually connected treatises on the three great departments into which modern physiology naturally divides itself." In accordance with this plan, the chapters on animal chemistry and on the structure and actions of the animal tissues, which occupied about 240 pages of the last edition, have been entirely omitted in the present, and the student who desires information on this important subject must look elsewhere for it, if his means permit of his obtaining the more expensive works in which it may be found, or—go without it. This omission, we feel constrained to say, we consider a capital mistake, and one which we think must eventually limit the circulation of the "Human Physiology" to the more advanced student and practitioner, and bring his "Elements" more prominently forward as a substitute in the hands of those engaged in attendance upon lectures.

With this exception, the present edition cannot fail to sustain the enviable reputation obtained by previous ones. Dr. Carpenter's well known zeal and industry are fully shown in the care evinced in the remodelling of some of the chapters, and the additions of new material by which the sixth edition is fully brought up to the times. Everything appertaining to the science which has appeared since the last edition, has been subjected to his reducing crucible, and a complete digest is afforded to those whose opportunities of research are more restricted. The views of the author are too well known to our readers to make any analysis necessary; suffice it to say, that where additional light has been thrown upon any subject, making a change of opinion necessary, the author has not hesitated to express it; a frankness calculated to inspire confidence in his readers and to insure a faithful reflection of the present state of physiological science.

The editor's notes are both full and numerous. We have been especially interested in his remarks upon the origin and destination of fibrin in the animal economy, and with the theory of the cause of the heart's action, and commend them to the reader's attention. Among the additions we observe Prof. Dalton's excellent article upon the Gastric Juice, published in the American Journal of Medical Sciences; Mr. Joseph Jones' paper on Endosmose, read before the Philadelphia Academy of Natural Sciences, and Professor Jackson's philosophical, satisfactory, and we believe entirely novel explanation of the functions of the semi-circular canals of the ear, besides a number of other valuable American and foreign contributions to the science, which have passed unnoticed by the author. Unusual judgment is shown, in our opinion, in these selection and additions, and we are confident that those who read them will agree with us that they materially enhance the value of the work.

The publishers deserve great credit for the handsome style in which the volume is printed and illustrated.

---

*The Diagnosis of Surgical Cancer.* (*The Liston Prize Essay for* 1854.) By JOHN ZACHARIAH LAURENCE, Surgeon to the Northern Dispensaries; late House Surgeon to University College Hospital. London: John Churchill. 1855. pp. 77, in two chapters, with two plates.

We have read this short monograph with considerable interest and have been so much pleased with its mode of treating its important and difficult subject, that we regret our inability to give it more than a cursory notice.

It is modestly offered "rather as a rough sketch of the Pathology and Anatomy of Cancer (which may one day serve as a basis to future investigation), than as laying any pretensions to the character of a finished treatise." Regarding it in such a light, it certainly does great credit to the scientific and practical training of its author; and at the same time encourages us to look to the same source for a much more extended and conclusive examination of the whole question, to which it already forms so excellent a ground work.

Justly considering the diagnosis of a case to be more or less concerned in the consideration of all its points, he briefly discusses the diagnostic value of these, severally, according to the following scheme:

"I. ÆTIOLOGY. *a. Predisposing causes.* 1. Hereditary; 2. Personal; sex, age, previous health. *b. Exciting Causes.* II. SYMPTOMATOLOGY. *a. Special Previous History.* 1. Progress of growth; 2. Patient's general health during that progress. *b. Condition of patient when first brought under observation.* 1. Local Phenomena; (consistence and aspect of growth, pain, hemorrhage, discharge, &c.); 2. General Phenomena. (Cachexia, wasting, &c.) *c. Anatomy of growth.* 1. Obvious Characters; 2. Microscopic Characters."

In this discussion he avails himself, in the first place, of the labors of Lebert, Paget, Walshe, Velpeau and others, together with some of the older observers, as Wardrop and Hey; and in the second place, of his own individual observations.

The clinical aspect of the subject, including causes and symptoms, occupies the first of the two chapters. It is treated in a practical manner, and although on account of its brevity it is necessarily more suggestive than conclusive,—more likely to unsettle than determine mooted questions—it is valuable in this very way and furnishes many useful hints. The same thing, to some extent, may be said of the remarks upon the anatomical elements of diagnosis, which are the subject of inquiry in the second chapter. We have only room to quote the author's concluding estimate of the microscopic test as a means of diagnosis.

"1. That in the greater number of cases of cancerous tumors the so-called cancer-cell will be found.

2. That this form of cell is occasionally seen in growths manifestly innocent.

3. That, *vice versâ* (what is, however, less frequent), tumors anatomically innocent prove clinically malignant—that 'the cancer-cell is not the *sine quâ non* character of cancer.'

4. That the inferences drawn from the microscopic examination are not to be deduced from a few isolated cells that may have happened to strike the eye, but rather from the characters of all the cells, and of the field of view generally.

5. That the results afforded by the microscope must take an important but not an exclusive and overbalancing position in the series of data, which are to serve us as the premises for our conclusion."

---

*The Book of Prescriptions: containing* 2900 *Prescriptions, collected from the practice of the most eminent physicians and surgeons, English and foreign. Comprising also a Compendious History of the Materia Medica of all countries, alphabetically arranged, &c.* By HENRY BEASLEY. Philadelphia: Lindsay & Blakiston.

Mr. Beasley's book belongs to a class of works, which in their proper place and as remembrancers are exceedingly useful. There are few physicians who have not their favorite prescriptions for particular diseases or stages of disease; it is impossible it should be otherwise where the practice is large; it is well for all, therefore, to be provided with a work like the present, in which can be found under the head of each remedy the various methods of prescribing it, adopted by practitioners of authority in each instance. Dr. Beasley is evidently an industrious, well-informed man, in every way qualified to supply this want, and we take much pleasure in recommending his work to the profession.

"It will readily be imagined," says the author, "that the prescriptions are not all of equal merit, and the reader will find several opportunities of comparison, by which a considerable variety of opinion among medical practitioners, particularly in the matter of doses, has been forcibly illustrated. When it has been necessary to point out the mean between extremes of this nature, the editor has been assisted in his task by competent medical advice, and he hopes that a correct judgment has been thereby arrived at."

The following is a sample of the work:

ACIDUM HYDROCYANICUM. *Hydrocyanic or Prussic Acid.*

Hydrocyanic acid is a direct sedative, and so highly poisonous, that a single grain of the pure acid is sufficient to destroy life. The diluted acid, in medicinal doses, allays irritation, reduces the pulse, and lowers the sensibility of the nervous system. It is used to quiet irritable and spasmodic cough, to allay vomiting, and nervous palpitations, and to relieve pain and quiet the system in neuralgic, rheumatic, and other painful affections. *Externally*, it is used in lotions, to allay itching in some cutaneous diseases. *Inhaled*, it has been tried in some affections of the lungs.

The usual dose of acidum hydrocyanicum dilutum, L. is from 3 to 5 minims. That of the new DUB. Ph. is, perhaps, intended to be of the same strength, but its percentage of real acid is not stated. The process yields a product which is variable, but usually stronger than that of L. Acidum hydrocyanicum, E., is stronger than L., 3 minims of the former being equal to about 5 of the other. *Acidum Hydrocyanicum* (*Scheelii*) is often met with in prescriptions; but there is no standard strength for it, and it possesses no advantage over the pharmacopœia preparation. The average strength, as procured from various manufactures, seems to be about twice that of L. *Acidum Hydrocyanicum* of the United States and Prussian Pharmacopœias contains, like that of L., 2 per centum of real acid.

*Potassii Cyanidum* has the same properties and uses as hydrocyanic acid. Dose from one-eighth to one-fourth of a grain. The latter dose is equal to 5 minims of diluted Hydrocyanic acid, L.

℞ Acidi Hydrocyanici dil. ♏j.

Aquæ destillatæ, f℥vij.

Syrupi Aurantii, fℨj.

M. fiat haustus quaque secunda hora sumendus donec evanescent symptomata.

*In Gastric Irritability, Nervous Palpitation, &c.*—DR. NELIGAN.

℞ Acidi Hydrocyan. dil. ♏xij.
Aquæ destillatæ, f℥j.
Syrupi Papaveris, fℨiij.
Misce: capitat cochl. amplum secunda quaque horâ.

*In Consumptive Cough, &c.*—DR. GRANVILLE.

℞ Potassæ Bicarbonatis, gr. xv.
Cocci Cacti, gr. viij.
Aquæ destillatæ, f℥vj. Tere simul, cola, et adde.
Acidi Hydrocyanici dil. ♏x. Misce.
A tablespoonful to be taken when the cough is troublesome.

*In Hooping Cough of Children.*—DR. GRANVILLE.

℞ Misturæ Amygdalæ, f℥vss.

Acidi Hydrocyan. dil. fℨss.

Tincturæ Opii, fℨss.

Tinct. Lavand. comp. fℨij.

Misce: fiat mistura cujus sumantur cochl. ij. larga secundis vel tertiis horis.

*In Gastrodynia with Spasms.*—DR. COPLAND.

℞ Acidi Hydrocyanici, guttas v.
Aquæ Calidæ, q. s.

Inhale the vapor by means of a suitable apparatus three times a day, lying down an hour after each ; very gradually increase the dose to ten drops. After four or six weeks, give steel and quinine.

*In Hypertrophy of the Heart.*—DR. T. G. HARE.

---

*A Dictionary of Terms used in Medicine and the Collateral Sciences.* By RICHARD D. HOBLYN, A. M. OXON. *Revised, with numerous additions,* by ISAAC HAYS, M. D., &c. Philadelphia: Blanchard & Lea. 1855.

This excellent little dictionary, now in its sixth edition abroad, has been considerably enlarged and improved by the introduction into it, by the American editor, of a large amount of new matter, in which the student will find, besides many terms recently added to science, the names of our native medicinal plants, the formulæ for the officinal preparations, &c., &c.

The preface states that "the editor has availed himself of very many recent sources of information in preparing his additions, among which he would especially mention the *Expository Lexicon,* by Dr. R. G. Mayne ; *Medical Botany,* by the late Dr. R. E. Griffith ; the recent works of Carpenter, Paget, Owen, and Jones and Sieveking ; and the admirable *United States Dispensatory* of Professors Wood and Bache."

An additional inducement to the American student to purchase the work, is, that it has been made to conform with the latest edition of the United States Pharmacopœia.

We warmly commend it to our readers.

---

*The Transactions of the American Medical Association, instituted* 1847. Vol. VIII. Philadelphia: Printed for the Association by T. K. and P. G. Collins. 1855.

Our limits prevent us from doing more than acknowledging the receipt of the above volume. We shall take another opportunity of noting its contents.

